# Levodopa Challenge Test Predicts STN-DBS Outcomes in Various Parkinson's Disease Motor Subtypes: A More Accurate Judgment

**DOI:** 10.1155/2021/4762027

**Published:** 2021-10-21

**Authors:** Zijian Zheng, Zixiao Yin, Bohan Zhang, Houyou Fan, Dan Liu, Yuancheng Zhou, Jian Duan, Dongwei Zhou, Xi Wu, Guohui Lu

**Affiliations:** ^1^Department of Neurosurgery, The First Affiliated Hospital of Nanchang University, Nanchang, Jiangxi, China; ^2^The First Clinical Medical College of Nanchang University, Nanchang, Jiangxi, China; ^3^Department of Neurosurgery, Beijing Tiantan Hospital, Capital Medical University, Beijing, China; ^4^Department of Neurology, Qilu Hospital, Shandong University, Jinan, Shandong, China; ^5^Department of Neurosurgery, Changhai Hospital of the Second Military Medical University of Chinese PLA, Shanghai, China

## Abstract

**Background:**

The relationship between the levodopa challenge test (LDCT) and postoperative subthalamic nucleus-deep brain stimulation (STN-DBS) benefits is controversial in patients with Parkinson's disease (PD). We aim to evaluate the value of total levodopa response (TLR) and symptom levodopa response (SLR) in predicting postoperative improvement in different PD motor subtypes.

**Methods:**

Studies were split into a training set (147 patients) and a validation set (304 patients). We retrospectively collected data from 147 patients who received the Unified Parkinson's Disease Rating Scale- (UPDRS-) III and the Parkinson's Disease Questionnaire- (PDQ-) 39 evaluation. Patients were classified into tremor-dominant (TD), akinetic-rigid-dominant (AR), and mixed (MX) groups. Clinically important difference (CID) was employed to dichotomize DBS effects. For patients in each subtype group from the training set, we used the correlation and receiver operator characteristic (ROC) curve analyses to explore the strength of their relations. Areas under the curve (AUCs) were calculated and compared through the DeLong test. Results developed from the training set were applied into the validation set to predict postoperative improvement in different PD motor subtypes.

**Results:**

In the validation cohort, TLR significantly correlated with postoperative motor (*p* < 0.001) and quality of life (QOL) (*p* < 0.001) improvement in the MX group. The AUC between TLR and UPDRS-III (TU) is 0.800. The AUC between TLR and PDQ-39 (TP) is 0.770. An associated criterion in both TU and TP is around 50%. In the AR group, strong correlation was only found in SLR and PDQ-39 (SP) (*p* < 0.001). And the AUC of SP is significantly larger than that in TLR and PDQ-39 (TP) (*p* = 0.034). An associated criterion in SP is around 37%. No significant correlation was found in the TD group.

**Conclusions:**

We provide a more accurate judgment for LDCT. TLR strongly correlated with postoperative UPDRS-III and PDQ-39 improvement in MX patients. A TLR > 50% may indicate a higher possibility of clinically meaningful benefits from STN-DBS comparing to medication only. SLR can well predict QOL improvement in AR patients. Similarly, a SLR > 37% may indicate a higher possibility of clinically significant benefits from STN-DBS. LDCT provides limited information for TD patients.

## 1. Introduction

Parkinson's disease (PD) is a neurodegenerative disease with two main therapies of levodopa and deep brain stimulation (DBS). Typically, an acute levodopa challenge test (LDCT) is conducted before DBS surgery to screen potential beneficiaries. Levodopa response (LR) assessed by the Unified Parkinson's Disease Rating Scale- (UPDRS-) III has been regarded as the best outcome predictor for postoperative response to DBS [1]. The relation between the preoperative LR and postoperative DBS benefits has long been disputed [2]. Some authors found that the preoperative LR is a key predictor for outcomes of bilateral STN-DBS for advanced PD [3, 4], while others indicated that the significant correlations are only the result of statistical methods and primary assumptions [5]. There are reports that patients who do not have a 30%-or-greater LR do obtain satisfactory improvements after DBS surgery [6, 7]. The mismatch between levodopa and DBS responses can be more commonly observed in single-symptom-dominated (SSD) patients, such as tremor-dominated patients or rigidity-dominated patients [8]. For those patients, the effect of levodopa on the total UPDRS score can be less informative than that on particular symptoms [9]. The LR toward particular symptoms calculated by UPDRS subitems, which we termed as “symptom levodopa response (SLR)” to distinguish from total levodopa response (TLR), might better predict STN-DBS efficiency in a certain group of patients. We employed both the receiver operating characteristic (ROC) curve analysis and correlation analysis to explore the predictive value of LDCT in different PD motor subtypes. In addition, since single-center outcomes may not be well generalized to a large population, we further validated our results in an external validation set to enhance the credibility of the findings.

## 2. Material and Methods

### 2.1. Patients

We reviewed the electronic medical records of all PD patients who received bilateral STN-DBS between June 1, 2015, and June 1, 2019, in the First Affiliated Hospital of Nanchang University. Patients with complete baseline and 3-month follow-up data were included. The diagnosis of PD was in accordance with the United Kingdom PD Society Brain Bank Diagnostic Criteria [10]. Sex, age, age at onset, disease duration, duration of motor fluctuations, and medication were recorded by inquiring the case history. Hoehn-Yahr stage, UPDRS, PDQ-39, Hamilton depression rating scale (HAMD), and Hamilton anxiety rating scale (HAMA) values were assessed for all included patients under the guidance of movement disorder specialists. The ethics committee of the First Affiliated Hospital of Nanchang University approved the study protocol, and all patients or their families provided written informed consent.

### 2.2. Subtype Classification

Two methods were commonly used to classify PD patients. Jankovic et al. [11] divided patients into tremor-dominant (TD), postural instability and gait difficulty, and intermediate motor subtypes, and Lewis [12] divided patients into TD, akinetic-rigid-dominant (AR), and mixed (MX) motor subtypes. We adopted Lewis' method and did not choose Jankovic's method because UPDRS-II was involved in Jankovic's classification process, which will cause problems to the calculation of SLR since UPDRS-II was not included in the LDCT. To divide the patients into various subtype groups, we calculated a tremor score (TS) and an akinetic-rigid score (ARS) for each patient in line with the methods previously reported. The TS was defined as the mean value of the sum of UPDRS items 20 and 21. The SLR of TS is calculated according to UPDRS items 20 and 21. The ARS was defined as the mean value of the sum of UPDRS items 18, 19, 22, and 27-31; therefore, the SLR of ARS is calculated according to UPDRS items 18, 19, 22, and 27-31. A patient was classified as TD if his or her TS/ARS ≥ 2. Conversely, a patient was classified as AR if his or her TS/ARS ≤ 0.5. The remaining patients, with a TS/ARS between 0.5 and 2, were classified as MX type. The classification was conducted based on the UPDRS off-medication score.

### 2.3. Patient Management and Follow-Up

After eliminating contraindications and signing the informed consent, all patients received LDCT and STN-DBS. Levodopa, compound levodopa, and other anti-Parkinson's drugs were stopped 12 hours before the test, and dopaminergic receptor agonists were stopped 72 hours before the test. Patients were administered 1.5 times the levodopa equivalent dose of the first dose they take every morning, and the test drug is standard compound levodopa. The electrode implantation was operated as follows. A stereotactic head frame was installed before CT scanning, and the CT imaging was fused with MRI to locate the STN. The surgical path was determined by a surgical planning workstation. Craniotomy was performed under local anesthesia, and the DBS devices (Medtronic 3387/3389 or PINS 1101) were implanted after target refinement by microelectrode recording and intraoperative test stimulation. Implantable pulse generators were then placed in the subclavicular position under general anesthesia. The DBS devices were programmed one month after the surgery. The patients accepted a postoperative programming with little difference. The stimulation effect was measured 3 months after surgery by the UPDRS-III and PDQ-39. The improvement of motor symptoms was calculated in both the on-medication/on-stimulation state and the off-medication/on-stimulation state. Regarding improvements of quality of life (QOL), we did not distinguish between on-medication and off-medication because the PDQ-39 reflects the QOL in the past month.

### 2.4. Dichotomize STN-DBS Effects

To better explore the predictive value of LDCT on patient's postoperative states, we divided patients into marked-improved ones and fair-improved ones. For motor improvement, we employed minimal clinically important difference (MICID) based on UPDRS-III to determine whether a patient got clinically meaningful improvement after surgery. MICID was established in approximately 6 points for detecting minimal, but clinically pertinent, improvement for UPDRS-III [13]. Since on-medications and on-stimulation can best represent the patient's postoperative state, an at-least-six-point difference in the comparison of baseline UPDRS-III on-medications and postoperative UPDRS-III on-medications and on-stimulation indicated the patient improved markedly. Since PDQ-39 scores reflect the overall QOL in both on- and off-medication states, the calculated score difference would overestimate the real improvement between preoperative-on-state and postoperative-on-state. Thus, we employed a stricter criterion for detecting clinically substantial changes. Moderate clinically important difference (MOCID) was established as approximately 3.5 points, around two times of MICID in PDQ-39 [14]. Patients reached an at-least-four-point difference in PDQ-39 after surgery was regarded as marked-improved patients.

### 2.5. External Validation Set

Results developed from the training set were validated in an external validation set for further evaluation. 304 PD patients who received the levodopa challenge test before STN-DBS in the Changhai Hospital Affiliated to Navy Medical University were included as a validation set. We viewed the baseline and the 3-month follow-up data of the PD patients who underwent STN-DBS surgery and employed the aforementioned method to divide these patients into TD, AR, and MX motor subtypes. Patients were divided into marked-improved ones and fair-improved ones in a similar way as described in the training set.

### 2.6. Statistical Analysis

Continuous data were presented as the mean ± SD. Comparison among the three groups was conducted by one-way ANOVA. Pairwise comparisons were conducted by the Bonferroni test. ROC curve was normally employed in the diagnosis test, but its application is not limited to diagnostic analysis. Authors have used it to explore the correlation between patient satisfactory and scale scores [15, 16]. ROC curve can better demonstrate the strength of correlation and visualize outcomes. In our study, we employed both ROC curve analysis and correlation analysis in the training set and the validation set. The areas under the curve (AUCs) show how well the classifier can distinguish marked-improved patients from fair-improved ones. Besides, the DeLong test made it possible to compare the strength of correlation. Pearson's correlation was employed to calculate the correlation coefficient. A scatter plot with a fit line and 95% CI was shown. The result of the ROC curve analysis was presented as AUC (95% CI). An AUC > 0.75 indicates the classifier provides clinically meaningful discriminative ability [17]. The Youden indexes and associated LDCT criteria were reported only in ROC curves with an AUC > 0.75. The DeLong tests were performed to compare different AUCs. A 2-tailed *p* value of 0.05 was considered statistically significant for the comparisons. All statistical procedures were performed using MedCalc version 15.2 (MedCalc, Ostend, Belgium) and SPSS version 24 (IBM, Chicago, IL).

## 3. Results

### 3.1. Baseline Characteristics and Patient Improvement

Data from the preoperative assessments and postoperative follow-ups in the training set are shown in Table 1. Of the preoperative indices, age at onset, duration of motor fluctuation, Hoehn-Yahr stage, UPDRS-III off scores, LR for akinetic-rigid score, LEDD, PDQ-39, and HAMA scores were significantly different between the three groups. For postoperative indices, the UPDRS-III and PDQ-39 scores assessed 3 months after the surgery were significantly different between the three groups. Overall, patients in the AR group have longer disease duration and worse baseline conditions. Fifty-eight patients reached MICID in comparing the baseline UPDRS-III on-medications and the postoperative UPDRS-III on-medications and on-stimulation. Seventy-six patients reached MOCID in comparing the baseline PDQ-39 on-medications and the postoperative PDQ-39 on-medications and on-stimulation. Related data from the preoperative assessments and postoperative follow-ups in the validation set are shown in Table 2.

### 3.2. The Relationship of LDCT and STN-DBS Benefits in the Training Set Patients

**Figure 1:**
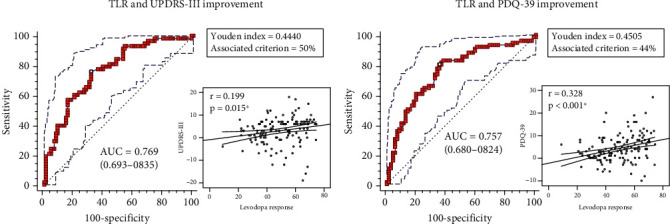
The receiver operating characteristic curves and scatter plots between TLR and postoperative STN-DBS benefits in the training set. (a) The combination of TLR and UPDRS-III improvement. (b) The combination of TLR and PDQ-39 improvement. Significant correlations are highlighted in bold and marked by ∗.

The ROC curves and scatter plots between preoperative LR and postoperative UPDRS-III and PDQ-39 improvement are shown in Figure 1. The AUC of LDCT in differentiating significant and insignificant motor beneficiaries is 0.769 according to UPDRS-III improvement. The AUC of LDCT in differentiating significant and insignificant QOL beneficiaries is 0.757 according to PDQ-39 improvement. The Youden indexes and their associated criteria are shown in the figure. Postoperative score changes of both UPDRS-III (*p* = 0.015) and PDQ-39 (*p* < 0.001) significantly correlate with preoperative levodopa response.

### 3.3. The Relationship of LDCT and STN-DBS Benefits in Different PD Subtypes

For the sake of clarity in the reporting, we used acronyms to represent the various classification and correlation combinations. TU represented the combination of TLR and UPDRS-III, and SP represented the combination of SLR and PDQ-39. Similarly, TP represented the combination of TLR and PDQ-39, and SU represented the combination of SLR and UPDRS-III.

#### 3.3.1. Classification Performance of SLR and TLR

**Figure 2:**
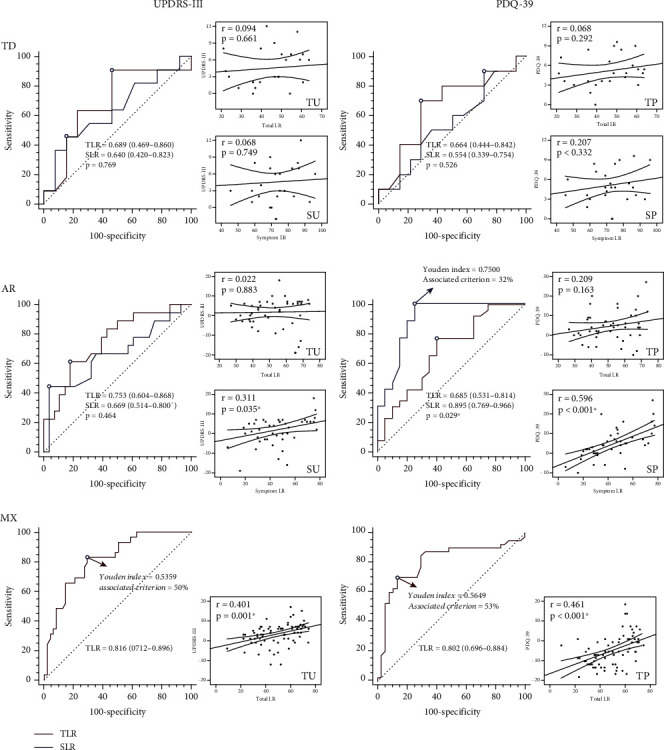
The receiver operating characteristic curves and scatter plots between TLR or SLR and postoperative STN-DBS benefits among the three PD motor subtypes in the training set. Significant correlations or comparisons are highlighted in bold and marked by ∗.

The ROC curves of different classification combinations are shown in Figure 2. No statistical difference was found between SLR and TLR in the TD group, while the AUC of SP is significantly larger than that of TP in the AR group (*p* = 0.029). In the MX group, the ROC of both TU (0.816) and TP (0.802) is above 0.8. The Youden indexes and associated LDCT criteria were reported if the AUC is above 0.75. For SP in the AR group, the Youden index is 0.75, and the associated criterion is 32%. For TU and TP in the MX group, the Youden index is 0.54 and 0.57, and the associated criterion is 50% and 53%.

#### 3.3.2. Correlation Performance of SLR and TLR

The scatter plots of different correlation combinations are shown in Figure 2. SLR positively correlated with both UPDRS-III (*p* = 0.035) and PDQ-39 (*p* < 0.001) improvement in the AR group. TLR positively correlated with both UPDRS-III (*p* < 0.001) and PDQ-39 (*p* < 0.001) improvement in the MX group. We found no significant correlation in the TD group.

### 3.4. Evaluation in the Validation Set

**Figure 3:**
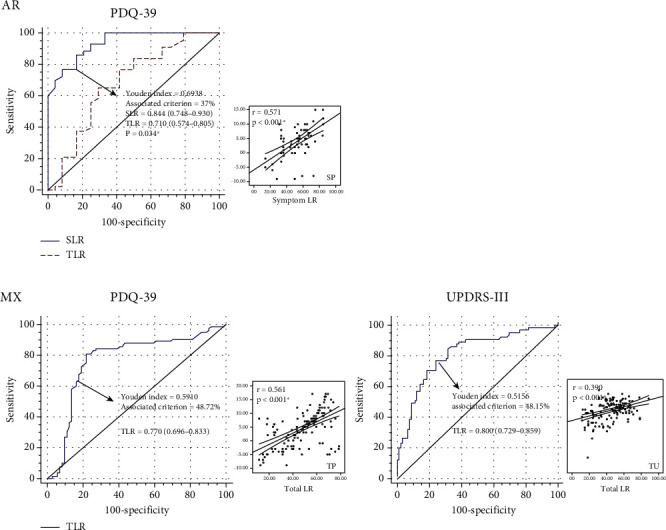
The receiver operating characteristic curves and scatter plots between TLR or SLR and postoperative STN-DBS benefits among the two PD motor subtypes in the validation cohort. Significant correlations or comparisons are highlighted in bold and marked by ∗.

ROC curves of different classification combinations are shown in Figure 3. In the MX group, the AUC of LDCT in TU is 0.800 with a Youden index of 0.52 and associated criterion of 48%. The AUC of LDCT in TP is 0.770 with a Youden index of 0.59 and associated criterion of 49%. In the AR group, the AUC of LDCT in SP is 0.844 with a Youden index of 0.66 and associated criterion of 37%.

## 4. Discussion

This study discussed the value of LDCT in predicting STN-DBS benefits in different PD motor subtypes and evaluated the findings in an external validation set. We found that on-state improvement is predictable by TLR, especially in MX patients. SLR strongly correlated with postoperative QOL improvement in AR patients. LDCT showed no significant predictive value in TD patients.

We employed both the methods of Pearson's correlation and the ROC curve to explore the relationship between LDCT and STN-DBS benefits. Pearson's correlation focused on detecting the consistency of two continuous variables while this method can only detect linearly correlated relations and is highly vulnerable to outliers [18]. Laying emphasis on exploring the predictive effect of continuous variables on binary variables, ROC curve analysis can alleviate the influence of outliers and can also show the strength of relation between two variables. Dichotomizing outcome variable according to research objective can endow associated ROC curve with different clinical significance. The construction of ROC curve is based on the classifier's sensitivity and specificity, which are both incidence measures, the percent or ratio of those patients who exceed a cutoff compared to those that did not reach cutoff. Consequently, establishment of the cutoff is very important. In our study, clinically important difference (CID) was used to differentiate marked-improved patients and fair-improved patients. CID has been widely employed in large clinical trial to reflect clinically meaning change [19]. Only around 40% and 50% of samples reached CID in UPDRS-III and PDQ-39 in our study, respectively. This is quite different from the ratio of 70% obtained by Katz et al. [20]. Possible reasons could be that we calculated on-state improvement while they calculated off-state improvement. The comparison between baseline UPDRS-III on-medications and postoperative UPDRS-III on-medications and on-stimulation better shows patients' overall improvement over medical treatment alone. This comparison is more clinically relevant to patients since this reflects the state that the patient is most likely to be in. Off-state comparison would be more helpful when only the stimulation effect is of interest.

The LDCT is commonly regarded as an important referee for predicting DBS effects. It helps to the diagnosis of PD, and typically, DBS response is more robust for the levodopa-responsive symptoms [21]. In the literature, several publications argued that preoperative LR does not predict long-term STN-DBS outcomes, but there are also reports claiming the contrary [2, 4]. In our study, in both the analysis in the training set and the validation set, the ROC curve showed that the predictive ability of LDCT on STN-DBS effects was not very solid since the AUCs were just over 0.75, despite the significant correlation being observed. However, the predictive ability increased a lot in the MX group both in the AUC and correlation coefficient *r*. The AUC of TLR in the MX group was the highest in all three subtypes. Possible reasons could be that the UPDRS-III score is more evenly distributed among all symptoms in the MX group. A uniform distribution could make the percent improvement of UPDRS-III in LDCT reflect the information of levodopa responsiveness more comprehensively. Instead, for SSD patients, the UPDRS-III score was mainly contributed by several subitems related to a certain symptom, while other less severe symptoms also have the same weight when calculating percentage improvement. This could result in that the calculated LR value does not match the real responsiveness to levodopa. Besides, MX patients have moderate baseline UPDRS scores, between that of TD and AR patients. An absence of outliers can also increase the accuracy of prediction. For MX patients, we further found that around 50% LR can predict STN-DBS effects. Approximately, patients with a LR > 50% are highly likely to gain clinically meaningful benefits from STN-DBS comparing to medication only. This is different from the widely accepted 30% LR. Thirty-percent LR is the clinically minimal motor improvement of UPDRS-III after taking a dopaminergic drug, which is aimed at assisting the diagnosis of PD through levodopa responsiveness [22]. However, many patients with LRs exceeding 30% do not get significant benefits from STN-DBS because this value was not originally established to predict DBS effects. In our study, 50% LR could well distinguish marked-improved patients and fair-improved patients only in the MX group. Interestingly, 50% is very close to the average postoperative UPDRS-III improvement reported by a large meta-analysis [23]. The strong correlation between LDCT and postoperative benefits in the MX group could possibly explain this.

As previously mentioned, for SSD patients, only one or two major symptoms are the source of most of their problems and are the target concerns they are most urgently willing to address. To better emphasize on the improvement of these dominated symptoms, we thus introduced the concept of SLR. However, we did not find any significant correlations between SLR or TLR and postoperative motor or QOL improvement in TD patients. The major reason could be that the responsiveness of severe tremor to levodopa therapy is not in accordance with that to the DBS therapy. In contrast, in the AR group, SLR showed strong correlation with PDQ-39 change. And the differentiating ability of SLR was significantly higher than TLR in judging QOL improvement. Regarding why QOL improvements are more predictable in the ARD group by SLR, we suspect the following reasons. First, patient expectation and satisfaction may play an important role here. Some patients may not receive significant benefit on total motor function, but addressing the problems of concern can greatly enhance their satisfaction and QOL score [7, 24]. SLR can accurately reflect improvements in major symptoms without being affected by the less-concerning items in UPDRS-III and thus can be more sensitive in judging a patient's possible QOL change [25]. Second, ARD symptoms, including gait disorder and postural instability, have long been reported as significant influencing factors upon QOL [26]. Gómez-Esteban et al. further indicated that rigidity had more impact on QOL than tremor [27]. The alleviation of annoying and dominant problem can undoubtedly increase patient's life quality. Besides, unlike tremor, which can be resistant to levodopa therapy, rigidity's responses toward levodopa and DBS are more consistent [28]. Third, the ARD patients in our study had the worst baseline conditions and PDQ-39 scores. And it is reported that patients with impaired preoperative QOL are more likely to have better postoperative QOL improvement [29]. For AR patients, we also found that patients with a SLR > 37% are more likely to gain clinically meaningful QOL benefits after STN-DBS. This will give us a reference in predicting postoperative QOL improvement even before the STN-DBS surgery.

Our study has several limitations. First, the data were retrospectively collected, and a relatively small sample size (only 24 patients in the TD group in the training set) could reduce statistical power. But it is generally harder to generate significant differences with a small sample size. Second, the follow-up period was short (3 months), leaving some long-term adverse events unrecognized including depression and progressive cognitive decline, which may also markedly affect QOL. Third, the data sources in our research only come from two single clinical centers, and it is necessary to carry out multicenter clinical research. Future studies should employ a prospective design, increase the sample size, and prolong the follow-up period to further strengthen the evidence, despite that an external verification was conducted in our study which will undoubtedly enhance the credibility of the findings.

## 5. Conclusion

We provide a more accurate judgment for LDCT. In a short follow-up period of three months, LDCT provides different information to the three PD motor subtypes receiving STN-DBS surgery. For MX patients, TLR is strongly correlated with postoperative motor and QOL improvement. A TLR > 50% may indicate a higher possibility of clinically meaningful benefits from STN-DBS. For AR patients, SLR can well predict postoperative QOL change. A SLR > 37% may indicate a higher possibility of clinically meaningful benefits from STN-DBS. Both TLR and SLR cannot provide valid predictive information for TD patients. These findings should be considered when screening PD-DBS candidates.

## Figures and Tables

**Table 1:** Comparison of baseline and postoperative indices among the three subtype groups in the training set.

	Total (*n* = 147)	TD (*n* = 24)	AR (*n* = 46)	MX (*n* = 77)	ANOVA^∗^	TD vs. AR^∗^	TD vs. MX^∗^	AR vs. MX^∗^
Sex (male/female)	87/60	10/14	29/17	48/29				
Reach MICID in UPDRS-III (*n*)	58 (39.5%)	11 (45.8%)	18 (39.1%)	29 (37.7%)				
Reach MOCID in PDQ-39 (*n*)	76 (51.7%)	10 (41.7%)	26 (56.5%)	40 (51.9%)				
Age at surgery (years)	62.3 ± 9.6	65.5 ± 6.8	59.9 ± 11.1	62.7 ± 9.1	0.053			
Age at onset (years)	52.8 ± 9.4	56.9 ± 6.5	49.9 ± 10.2	53.3 ± 9.3	**0.009**	**0.008**	0.295	0.131
DD (years)	9.5 ± 2.7	8.6 ± 1.6	10.0 ± 2.7	9.4 ± 2.8	0.106			
Duration of MF (years)	5.2 ± 2.8	3.7 ± 1.9	6.4 ± 2.8	5.1 ± 2.7	**<0.001**	**<0.001**	0.088	**0.030**
H-Y on	2.2 ± 0.8	1.8 ± 0.9	2.4 ± 0.9	2.1 ± 0.8	**0.007**	**0.005**	0.128	0.239
H-Y off	3.2 ± 1.1	2.7 ± 1.3	3.4 ± 0.9	3.2 ± 1.1	**0.049**	**0.043**	0.219	0.843
UPDRS-III on	17.8 ± 6.1	17.0 ± 5.8	19.4 ± 7.0	17.0 ± 5.5	0.102			
UPDRS-III off	37.9 ± 15.2	32.7 ± 12.4	42.3 ± 15.5	37.0 ± 15.2	**0.030**	0.381	1.000	0.126
LR for UPDRS-III (%)	49.4 ± 15.5	45.5 ± 13.1	51.7 ± 13.1	49.3 ± 15.6	0.230			
LR for tremor score (%)	77.1 ± 17.9	72.2 ± 13.2	74.9 ± 16.9	80.0 ± 19.4	0.107			
LR for akinetic-rigid score (%)	42.1 ± 18.7	25.1 ± 21.3	44.1 ± 18.6	46.3 ± 14.8	**<0.001**	**<0.001**	**<0.001**	1.000
LEDD	987 ± 306	915.6 ± 356.2	1089.7 ± 297.3	949.3 ± 283.4	**0.021**	0.068	1.000	**0.040**
PDQ-39	35.8 ± 11.6	28.3 ± 9.7	39.9 ± 11.9	35.7 ± 10.9	**<0.001**	**<0.001**	**0.014**	0.135
HAMD	12.7 ± 3.6	12.4 ± 2.9	13.3 ± 3.7	13.8 ± 2.6	0.150			
HAMA	11.9 ± 3.4	11.6 ± 2.9	13.4 ± 2.9	12.5 ± 2.6	**0.025**	**0.027**	0.527	0.187
Post UPDRS-III on	14.7 ± 6.8	12.5 ± 4.4	17.6 ± 8.2	13.7 ± 6.0	**0.002**	**0.007**	1.000	**0.006**
Post UPDRS-III off	19.9 ± 8.7	16.7 ± 5.7	23.4 ± 10.3	18.8 ± 7.8	**0.002**	**0.005**	0.813	**0.012**
Post PDQ-39	31.6 ± 9.8	24.8 ± 7.9	35.2 ± 10.1	31.6 ± 9.2	**<0.001**	**<0.001**	**0.006**	0.120

TD: tremor-dominated patients; AR: akinetic-rigid-dominated patients; MX: mixed patients; MICID: minimal clinically important difference; MOCID: moderate clinically important difference; DD: duration of diagnosis; MF: motor fluctuation; H-Y: Hoehn-Yahr stage; UPDRS: Unified Parkinson's Disease Rating Scale; LR: levodopa response; LEDD: levodopa equivalent doses; PDQ-39: Parkinson's Disease Questionnaire-39; HAMD: Hamilton depression scale; HAMA: Hamilton anxiety scale; Post: postoperative; Post UPDRS-III on: on-stimulation/on-medicine; Post UPDRS-III off: on-stimulation/off-medicine. ^∗^*p* value. Significant comparisons are highlighted in bold.

**Table 2:** Comparison of baseline and postoperative indices among the three subtype groups in the validation set.

	Total (*n* = 304)	TD (*n* = 80)	AR (*n* = 67)	MX (*n* = 157)	ANOVA^∗^	TD vs. AR^∗^	TD vs. MX^∗^	AR vs. MX^∗^
Sex (male/female)	157/147	43/37	32/35	82/75				
Reach MICID in UPDRS-III (*n*)	145 (47.7%)	35 (43.8%)	45 (67.1%)	65 (41.4%)				
Reach MOCID in PDQ-39 (*n*)	76 (51.7%)	41 (41.7%)	32 (47.8%)	82 (52.2%)				
Age at surgery (years)	60.7 ± 7.6	63.5 ± 5.7	61.2 ± 10.7	60.8 ± 7.3	0.178			
Age at onset (years)	55.4 ± 6.4	52.9 ± 5.5	48.3 ± 7.2	50.3 ± 5.5	**0.003**	**<0.001**	0.178	0.232
DD (years)	8.5 ± 2.4	8.6 ± 1.8	9.1 ± 1.5	7.8 ± 3.4	0.235			
Duration of MF (years)	6.2 ± 2.1	3.5 ± 2.1	7.3 ± 3.1	4.3 ± 2.0	**<0.001**	**<0.001**	0.234	**0.015**
H-Y on	1.8 ± 1.0	2.3 ± 0.9	1.7 ± 1.0	3.5 ± 0.4	**0.012**	**0.123**	0.228	**0.022**
H-Y off	4.2 ± 1.1	3.3 ± 1.8	3.1 ± 1.1	3.3 ± 1.5	0.172	0.243	0.919	0.843
UPDRS-III on	19.3 ± 7.1	18.0 ± 4.8	17.4 ± 6.0	18..0 ± 5.7	0.202			
UPDRS-III off	36.6 ± 14.8	33.5 ± 11.3	41.3 ± 14.6	38.6 ± 17.3	**0.043**	0.381	1.000	0.126
LR for UPDRS-III (%)	50.2 ± 13.5	47.3 ± 12.8	51.3 ± 12.1	53.3 ± 13.8	0.371			
LR for tremor score (%)	75.2 ± 16.9	74.9 ± 11.8	75.4 ± 15.2	79.0 ± 20.1	0.207			
LR for akinetic-rigid score (%)	41.5 ± 17.7	24.9 ± 20.7	43.2 ± 16.6	45.4 ± 13.9	**<0.001**	**<0.001**	**<0.001**	0.945
LEDD	955 ± 321	873.3 ± 342.2	989.7 ± 286.3	903.3 ± 222.4	**0.015**	0.023	0.847	**0.030**
PDQ-39	34.7 ± 10.6	29.3 ± 7.7	37.5 ± 12.9	34.4 ± 8.9	**<0.001**	**<0.001**	**0.026**	0.365
HAMD	10.7 ± 3.6	11.4 ± 3.9	12.3 ± 3.3	12.8 ± 3.6	0.333			
HAMA	9.9 ± 2.7	10.3 ± 1.9	12.4 ± 1.9	11.5 ± 1.7	**0.013**	**0.019**	0.433	0.636
Post UPDRS-III on	13.5 ± 7.8	11.1 ± 5.4	16.6 ± 8.2	12.5 ± 5.0	**0.003**	**0.002**	0.936	**0.012**
Post UPDRS-III off	18.5 ± 7.7	14.7 ± 5.7	21.2 ± 9.3	16.3 ± 6.9	**0.002**	**<0.001**	0.623	**0.008**
Post PDQ-39	30.3 ± 8.9	25.2 ± 7.3	34.3 ± 8.1	28.9 ± 7.3	**<0.001**	**<0.001**	**0.023**	0.078

TD: tremor-dominated patients; AR: akinetic-rigid-dominated patients; MX: mixed patients; MICID: minimal clinically important difference; MOCID: moderate clinically important difference; DD: duration of diagnosis; MF: motor fluctuation; H-Y: Hoehn-Yahr stage; UPDRS: Unified Parkinson's Disease Rating Scale; LR: levodopa response; LEDD: levodopa equivalent doses; PDQ-39: Parkinson's Disease Questionnaire-39; HAMD: Hamilton depression scale; HAMA: Hamilton anxiety scale; Post: postoperative; Post UPDRS-III on: on-stimulation/on-medicine; Post UPDRS-III off: on-stimulation/off-medicine. ^∗^*p* value. Significant comparisons are highlighted in bold.

## Data Availability

The relevant valuable data are described in the manuscript.

## Abbreviations

PD: Parkinson's disease
STN-DBS: Subthalamic nucleus-deep brain stimulation
LDCT: Levodopa challenge test
LR: Levodopa response
TLR: Total levodopa response
SLR: Symptom levodopa response
SSD: Single-symptom-dominated
UPDRS: Unified Parkinson's Disease Rating Scale
PDQ-39: Parkinson's Disease Questionnaire-39
HAMD: Hamilton depression rating scale
HAMA: Hamilton anxiety rating scale
QOL: Quality of life
TS: Tremor score
ARS: Akinetic-rigid score
TD: Tremor-dominant
AR: Akinetic-rigid dominant
MX: Mixed
CID: Clinically important difference
MICID: Minimal clinically important difference
MOCID: Moderate clinically important difference
ROC: Receiver operating characteristic
AUC: Area under the curve
TU: TLR~UPDRS-III
TP: TLR~PDQ-39
SU: SLR~UPDRS-III
SP: SLR~PDQ-39.

## Additional Points

*Highlights*. TLR and SLR can predict postoperative improvement in different PD motor subtypes. TLR correlated with postoperative motor and quality of life improvement in the MX group. A TLR > 50% may indicate a higher possibility of clinically meaningful benefits from STN-DBS in the MX group. SLR correlated with postoperative motor and quality of life improvement in AR patients. A SLR > 37% may indicate a higher possibility of clinically meaningful benefits from STN-DBS in AR patients.
